# When Do Antidepressants “Kick In”: Addressing the 2–4-Week Myth

**DOI:** 10.1177/07067437261449898

**Published:** 2026-05-07

**Authors:** Stefani Mihilli, Anthony Levitt, Marco Solmi, Mark Sinyor, Ayal Schaffer

**Affiliations:** 1 Department of Psychiatry, 12366 Temerty Faculty of Medicine, University of Toronto, Toronto, Ontario, Canada; 2 Department of Psychiatry, 71545 Sunnybrook Health Sciences Centre, Toronto, Ontario, Canada; 3 SCIENCES lab, Department of Psychiatry, 12365 Faculty of Medicine, University of Ottawa, Ottawa, Ontario, Canada; 4 Department of Mental Health, 10055 The Ottawa Hospital, Ottawa, Ontario, Canada; 5 Department of Child and Adolescent Psychiatry, 7938 Ottawa Hospital Research Institute (OHRI), Ottawa, Ontario, Canada; 6 Department of Child and Adolescent Psychiatry, 14903 Charité Universitätsmedizin, Berlin, Germany

**Keywords:** depressive disorder, antidepressants, psychopharmacology

Treatment guidelines for major depressive disorder (MDD) recommend pharmacotherapy as the first-line treatment for many patients with depression. Prior to initiating treatment with an antidepressant medication, patients are counselled regarding the expected onset of clinical improvement. Yet, there is a substantial mismatch between published recommendations surrounding what to tell patients and the precise interpretation of evidence regarding the time to onset of antidepressants. According to the American Psychiatric Association guidelines (2010), it may take 2–4 weeks before patients may notice the beneficial effects of pharmacotherapy.^1^ Similarly, the UK National Institute for Health and Care Excellence depression guidelines (2022) encourage providers to advise patients that if the medication is efficacious for them, they can expect improvements within 4 weeks.^2^

**Figure 1.**
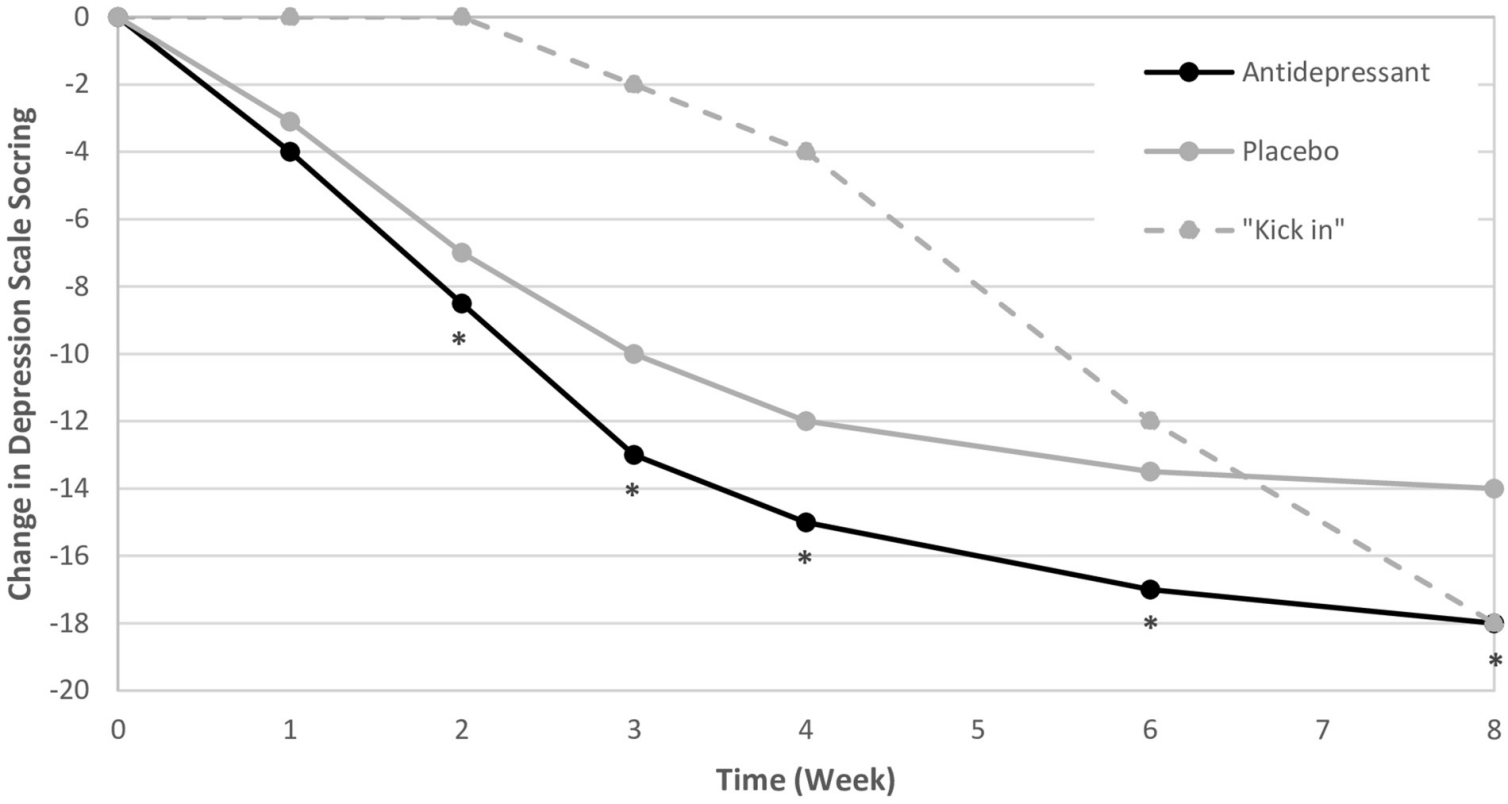
Illustrative example of a typical placebo-controlled antidepressant RCT for major depression.

Randomized controlled trials (RCTs) that investigate the onset of antidepressant action reveal improvements over time that generally resemble illustrative Figure 1. This is a conceptual figure created to reflect typical results from placebo-controlled RCTs in MDD investigating clinical improvement with antidepressants. In general, clinical improvement that is measured through a validated scale may enhance identification of early improvement, and more data is needed during these early stages to better characterize the specific course of early antidepressant response. In these studies, the onset of action is often reported as the time at which the antidepressant arm reaches a statistically significant difference from the placebo arm. In further examining these data, the figure highlights that the first 2 weeks of active antidepressant treatment are when the greatest rate of change on the depression rating scale occurs, with change reaching a plateau around the 6- to 8-week mark. Clinically, this means that, in general, patients may experience the most rapid improvement in their depressive symptoms early in the treatment course. This is further supported by a meta-analysis, which reported that one third of the total symptomatic improvement observed with SSRIs during 6-week treatment was observed within the first week.^3^ This is distinct from the “2–4-week” timeline and even more so from the classic “4–6 weeks” provided to patients as the expectant onset of antidepressant action.

Why the difference? We argue that the somewhat misleading guidance that has been common practice for physicians for decades arises from a misinterpretation of the clinical trial findings. This misinterpretation focuses on the time period in which a statistically significant difference emerges in the symptom reduction between the active treatment and placebo arms. This time point is highly germane when establishing group differences in clinical trials, but not necessarily at the individual patient level. From the perspective of an RCT that is aiming to establish whether an active treatment is efficacious, the timing of the appearance of a statistically significant delta between antidepressant and placebo arms is a key metric of interest. Regulatory trials are typically 4 to 6 weeks in length, which makes sense, since this is the period in which a significant separation is typically observed.

However, from a clinical and psychoeducational perspective, the key element of clinical treatment is the change experienced by the person taking the antidepressant. What patients wish to know is, “When will I start to feel better?” Clinically, the focus is on the patient's experience of improvement over time, which is fundamentally distinct from a statistical finding indicating separation of active treatment from placebo. From a patient experience perspective, therefore, the most salient information from an RCT is the trajectory of change within the antidepressant arm. This is because these arms approximate the impact of actual clinical care, which includes the combination of both specific benefits arising through exposure to the active antidepressant and non-specific benefits of participating in and receiving comprehensive clinical care that are captured under “placebo effects.” Placebo effects encompass positive elements of standard clinical care; they should not be “subtracted” from the overall understanding of how patients respond to receiving antidepressants and can be utilized by clinicians to promote hope and expectancy of response.

For these reasons, clinicians should educate patients that, on average, they are likely to experience early benefit as indicated by the black line in Figure 1. In contrast, typical counselling suggests to patients that antidepressants typically “kick in” at weeks 2–4, or even later, a pattern of change that is substantially different from what is actually observed in RCTs and more in line with the hypothetical dashed line in Figure 1.

Telling patients that antidepressants “kick in” after 2–4 weeks is not only potentially inaccurate but also may cause unnecessary distress or even reduce positive outcomes. There is strong evidence that patients’ expectations can partially mediate the effects of antidepressant treatment, and that counselling that is delivered in a way that enhances patient expectations regarding response can be beneficial.^4^ As such, by providing information that does not align with the patient experience of the time course of onset, clinicians may inadvertently demoralize patients who are already experiencing a time of great suffering and interfere with positive expectations that often help facilitate a more rapid response.

If RCTs consistently identify that initial improvement of symptoms generally occurs earlier than 2–4 weeks, then this should be communicated openly to patients. Guidance regarding communication to patients should rely less upon group differences from placebo and more upon individual patient experience. Importantly, this approach to understanding the onset of effect is distinct from predicting a patient's ultimate response vs. non-response to medication, the timing of establishing non-response, and any subsequent changes in medication management. Further nuances in the interpretation of antidepressant RCT data also exist, depending on the subtypes and dosing of antidepressants and the ways in which these interact with individual patient experience on medication. While RCT data reflect average scores over time, they do not generally report individual trajectories of response. Further studies would benefit from more precise exploration to quantify the degree to which specific antidepressant classes and dosing strategies may mediate the onset and trajectory of response. Such evidence would assist in further refining the guidance provided to patients receiving antidepressant medications. Overall, patients deserve to know that they may feel better sooner, and by effectively communicating this, we fulfill our role as medical experts, provide accurate information, and foster the hope that our patients deserve.

Clinical Pearls:

- The onset of action of antidepressants is being conflated with the time at which the antidepressant arm reaches a statistically significant difference from the placebo arm in RCTs and is being reported to patients as such.
- Antidepressant medications do not have a discrete “kick in” period in MDD; rather, patients often experience substantial symptom improvement within the first 2 weeks of treatment, and that clinical onset of improvement may start to build very soon after the medication is started, due to a combination of biological and non-specific placebo-type effects.
- Example of **current** counselling that patients receive regarding antidepressant onset: “Antidepressants usually take 2–4 weeks for their effect to kick in.”
  Example of **proposed** counselling for patients regarding antidepressant onset:
  “It is very possible that you may begin to notice some improvements in your symptoms soon, even within days, after starting your antidepressant, and that this will build up over time.”
